# Downregulation of Lysosomal Acid Ceramidase Mediates HMGB1-Induced Migration and Proliferation of Mouse Coronary Arterial Myocytes

**DOI:** 10.3389/fcell.2020.00111

**Published:** 2020-03-10

**Authors:** Xinxu Yuan, Owais M. Bhat, Hannah Lohner, Yang Zhang, Pin-Lan Li

**Affiliations:** ^1^Department of Pharmacology and Toxicology, School of Medicine, Virginia Commonwealth University, Richmond, VA, United States; ^2^Department of Pharmacological and Pharmaceutical Sciences, College of Pharmacy, University of Houston, Houston, TX, United States

**Keywords:** ceramide, cell migration, cell proliferation, lysosomes, coronary artery

## Abstract

High-mobility group box 1 protein (HMGB1) has been reported to trigger lysosome destabilization causing a wide of inflammatory diseases. The present study tested whether a lysosomal enzyme, acid ceramidase (AC), plays a critical role in HMGB1-induced alteration in ceramide metabolism and whether such HMGB1-AC interaction is associated with abnormal migration and proliferation of vascular smooth muscle cells (SMCs). We first observed that the expression of AC in the medial layer of mouse coronary arterial wall and colocalization of AC with a lysosome marker Lamp-1. In primary cultured coronary arterial myocytes (CAMs), AC expression and colocalization with Lamp-1 were significantly up-regulated by AC inducer, genistein, but down-regulated by AC inhibitor, N-oleoylethanolamine (NOE). HMGB1 dose-dependently decreased the colocalization of AC with Lamp-1 and reduced mRNA and protein expressions of AC in CAMs, but reversed by genistein. Consistently, HMGB1 significantly induced increases in the levels of long-chain ceramides in CAMs, which were not further enhanced by NOE but blocked by genistein. More importantly, HMGB1 promoted migration and proliferation of CAMs, which were not further increased by NOE but reduced by genistein. Lastly, CAMs isolated from smooth muscle-specific AC knockout mice (AC gene *Asah1*) exhibited increased ceramide levels and enhanced the migration and proliferation, which resembles the effects of HMGB1 on wild-type CAMs. Together, these results suggest that HMGB1 promotes SMC migration and proliferation via inhibition of AC expression and ceramide accumulation.

## Introduction

High-mobility group box 1 protein (HMGB1) was originally identified as a ubiquitous non-histone DNA-binding nuclear protein and cytosolic protein produced by almost all types of injured or necrotic cells such as mouse smooth muscle cells (SMCs)(Kim et al., 2018; Wang et al., 2018; Li et al., 2019), endothelial cells (Jiang and Steinle, 2018; Xie et al., 2018; Ma et al., 2019), hepatocytes (Zhao et al., 2017; Zhang et al., 2019), and macrophage cells (Dragomir et al., 2011; Irie et al., 2017; Elfeky et al., 2018). HMGB1 serves as an important inflammatory mediator involved in the pathogenesis of inflammatory diseases such as sepsis (Stevens et al., 2017), rheumatoid arthritis (Park et al., 2015), atherosclerosis (Andrassy et al., 2012), and aneurysm (Kohno et al., 2011, 2012; Ousaka et al., 2017). Nuclear HMGB1 and cytosolic HMGB1 have distinct functions. The nuclear HMGB1 is involved in gene transcription and chromatin structure, whereas the cytosolic HMGB1 mediates the inflammasome activation and autophagy (Jiang and Pisetsky, 2008). It is known that extracellular HMGB1 binds to the receptor for advanced glycation end products (RAGE), Toll-like receptor (TLR)2 as well as TLR4 and TLR9 and other related cell signaling transduction receptors (Tsung et al., 2014), and consequently causes pro-inflammatory responses through secretion of pro-inflammatory cytokines (Andersson et al., 2000). Many specific (anti-HMGB1 antibodies, glycyrrhizin) or unspecific (soluble RAGE, ethyl pyruvate) agents have been used in various experimental models to ameliorate the inflammation with improved outcomes. However, these anti-inflammatory therapy lacks efficacy in a number of preclinical models including acute and chronic inflammatory diseases regulated by infectious or sterile agents. Thus, it is of interest to investigate whether HMGB1 causes cell injury or dysfunction in addition to its inflammatory effects.

Recently, extracellular HMGB1 was reported to complex with more different inflammatory molecules endocytosed to the endolysosomal system upon binding to RAGE (Andersson et al., 2018a). The internalized HMGB1 destabilizes the lysosomal membrane and initiates a cascade of molecular events, including cathepsin B activation and release from ruptured lysosomes leading to pyroptosome formation and caspase-1 activation, which induces inflammation by providing access for HMGB1-partner molecules to cytosolic receptors (Xu et al., 2014). Thus, the lysosomal function disturbance might be associated with inflammation induced by HMGB1. In addition to inflammation induction, lysosome destabilization and impaired autophagy are linked with increased phenotypic switching of vascular SMCs from a quiescent “contractile” phenotype to a “synthetic” phenotype with enhanced proliferative and migratory potential (Salabei and Hill, 2015). Thus, HMGB1 may also induce lysosome injury and cause non-inflammatory effects such as phenotypic modulation of SMCs.

Ceramide is generated through hydrolysis of membrane sphingomyelin by sphingomyelinases such as acid sphingomyelinase (ASM) or neutral sphingomyelinase (NSM) or through *de novo* ceramide synthesis by serine palmitoyltransferase (SPT) and ceramide synthase (Futerman and Hannun, 2004; Boini et al., 2011). In lysosomes, ceramide can be further metabolized by acid ceramidase (AC) into sphingosine, which is further converted to sphingosine 1-phosphate (S1P) by sphingosine kinase (Boini et al., 2010). AC deficiency results in ceramide accumulation, which leads to a spectrum of disorders such as Farber disease, a rare lysosomal storage disorder, and spinal muscular atrophy with progressive myoclonic epilepsy, a rare epileptic disorder (Yu et al., 2018a). AC deficiency is also associated with Alzheimer’s, diabetes, and cancer (Gebai et al., 2018). Previous studies have shown that ceramides serve as an intracellular second messenger to mediate SMC migration (Mu et al., 2009; Liao et al., 2010), proliferation (Chatterjee, 1998; Mu et al., 2009; Liao et al., 2010), or calcification (Liao et al., 2013; Song et al., 2017). However, it remains unknown whether HMGB1 inhibits the AC expression or its function in SMCs and whether the functional loss of this lysosomal enzyme by HMGB1 causes ceramide accumulation contributing to SMC migration and proliferation.

The present study aimed to test the hypothesis that AC inhibition mediates HMGB1-induced proliferation and migration in SMCs. We first characterized the expression of AC in the mouse primary cultured coronary arterial myocytes (CAMs) or in the mouse coronary arterial wall. We then examined the effects of pharmacological intervention with AC or genetic deficiency of AC gene *Asah1* on HMGB1-induced changes in ceramide levels in CAMs and their cell proliferation and migration.

## Materials and Methods

### Isolation and Culture of Mouse Coronary Arterial Myocytes (CAMs)

As previously described, mouse CAMs were isolated (Adhikari et al., 2015). Briefly, the mice were anesthetized by 2% isoflurane. Then, the coronary arteries in the heart were isolated under a microscope and placed into phosphate-buffered saline (PBS) and kept on ice. Using angled forceps under a microscope, the adventitia was removed from the artery and immediately washed 2–3 times with PBS. The tissue was spread with forceps and rinsed to remove endothelial cells. Using micro-dissecting scissors in a cell culture hood, the tissues were cut into approximately 1–2 mm of pieces using micro-dissecting scissors. Then, the dissected tissues were rinsed with FBS and transferred into a flask for 2 h in a humidified 5% CO2, 37°C incubator. After 2 h, 10% FBS (Invitrogen, FBS001-HI, United States) and 2% antibiotics supplemented with Dulbecco’s Modified Eagle Medium (DMEM) (Thermo Fisher, 11995073, United States) was added to the flask. 1 week later, CAMs cloning was conducted by selecting cells from cell growing islands in the dish. Passage 3–8 of CAMs were used for *in vitro* study. Before studying, CAMs were identified and purified as reported previously (Xu et al., 2012). This study was performed following the principles of the Basel Declaration and recommendations in the Guide for the Care and Use of Laboratory Animals of the National Institutes of Health. The animal protocol was approved by the Institutional Animal Care and Use Committee (IACUC) at Virginia Commonwealth University.

### Western Blot Analysis

Specific proteins were analyzed using the protocol reported previously (Mo et al., 2018). 2 × 10^5^ of CAMs were seeded in 6-well plates overnight and treated with HMGB1 (0.4 ug/mL), Genistein (40 nM) or NOE (25 uM) for 24 h. Treated CAMs were homogenized in 30 μl of RIPA lysis buffer for 30 min on ice. Using Bio-Rad Protein Assay Dye (Bio-rad,500006, United States) to measure total protein and normalize to 1 μg/ml. After loading 15–20 μg of protein into the wells of a 12% SDS-PAGE gel, it was run for 3 h at a voltage of 100 V. The gel was transferred to nitrocellulose membranes (Millipore, IPVH00110, United States) at 100 V for 1–2 h in the cold room. The non-specific proteins were blocked using 5% non-fat milk (Bio-Rad, 1706404, United States) in Tris-buffered saline with Tween-20 (TBST) buffer for 1 h at room temperature. The blot was incubated with primary antibodies overnight at 4°C. The first antibodies used for Western blot analysis were AC (1:1000, Santa Cruz sc-28486, United States) and β-actin (Santa Cruz, SC-47778, United States). The second antibody labeled with HRP was incubated for 1 h at room temperature. Odyssey FC Imaging system was used to detect the bands. The intensity of the specific proteins was measured with Image J 6.0 (NIH, Bethesda, MD, United States) or Odyssey software.

### Immunofluorescence Staining

Frozen heart sections or CAMs cultured on cover slides were fixed with 4% paraformaldehyde (PFA) for 10–15 min on ice. Samples were treated in 0.1% Triton X-100 in PBS for 10 min at room temperature and incubated overnight at 4°C with the following primary antibodies: AC (1:200, Santa Cruz sc-28486, United States); lysosome marker, and anti-lamp-1 antibody (1:500, Abcam, ab25245). After washing 2–3 times with PBS, samples were incubated with a second antibody, labeled with either Alexa-555 or Alexa-488, for 1 h at room temperature in the darkroom. The images were captured using a confocal laser scanning. Image J 6.0 (NIH, Bethesda, MD, United States) was used to quantify the intensity of the cells and tissues. The colocalization was measured with Image-Pro Plus version 6.0 software (Media Cybernetics, Bethesda, MD). Pearson correlation coefficient (PCC) represented the colocalization of different proteins as previously reported (Chen et al., 2018).

### Immunohistochemistry

The wax slides were heated for 20 min on 65°C hot plate. Deparaffinization was performed for 10–15 min in 100% xylene. Rehydration was conducted in the graded 100%, 95%, and 75% ethanol. Antigen retrieval was performed in the citric acid buffer (pH 6.0) for 10 min at 98°C. 3% H_2_O_2_ in methanol was used to quench the endogenous peroxidase activity. 2% of horse serum, used to block non-specific proteins, was incubated for 1 h at room temperature. Primary antibodies AC (1:1000, Santa Cruz sc-28486, United States) was incubated overnight at 4°C. After 2–3 washes with PBS, biotinylated secondary antibodies and a streptavidin-peroxidase complex were used to incubate sections for 20 min at room temperature. The sections were then sequentially developed with fresh 3, 3-Diaminobenzidine (DAB) solution for 1 min. The sections were put into hematoxylin for 3 min and dehydrated in graded 75%, 95%, 100% ethanol for 5 min. Finally, slides were mounted with DPX as previously reported (Bhat et al., 2015).

### mRNA Expression Analysis

Real-time RT-PCR was conducted to detect mRNA expression as described previously (Xu et al., 2015). 2 × 10^5^ of CAMs were seeded in 6-well plates overnight and treated with different concentration of HMGB1 (0–1 ug/mL), Genistein (40 nM) or NOE (25 uM) for 24 h. Briefly, TRIzol RNA Kit (Thermo Scientific, 15596026, United States) was used to extract the total RNA. 1 μg of RNA was synthesized to cDNA using Bio-Rad cDNA Synthesis Kits. The PCR reaction was conducted using optimized SYBR^®^ Green qPCR mixture buffer. To normalize the expression of mRNA, β-actin was applied and the relative change was calculated by 2^–ΔΔ*Ct*^. The primers were designed by Operon (Huntsville, AL, United States) and the sequences were synthesized as follows: AC forward 5′- ACAACTGTGTAGGATTCACGCATTCTCC -3′;AC reverse, 5′-TCGATCTATGAAATGTCGCTGTCGG-3′, β-actin forward 5′-TCGCTGCGC-TGGTCGTC-3′; β-actin reverse 5′-GGCCTCGTCACCCACATAGGA-3′.

### High-Performance Liquid Chromatography-Tandem Mass Spectrometry (HPLC-MS/MS) Analysis of Ceramides

Quantitation of ceramides was measured using HPLC-MS/MS (Hong et al., 2019). Cells were treated using same condition with Westen blot and PCR experiment. 10 ng of each ceramide: C14, C16, C18, C20, C22, C24, was added to each sample and calibrator as the internal standard solution. Bligh-Dyer method was used to extracted ceramides under acidic conditions. Briefly, 1 N HCl and 2:1 of methanol: chloroform (v/v) was mixed with calibrator and each sample. After being sonicated for 30 s, the samples were mixed with calibrators. Then the samples were stood on the rack for 5 min at room temperature and centrifuged for 5 min at 13,200 rpm. The organic phases were transferred to the new tubes and extracted again with 100% chloroform. After being dried using nitrogen gas, the samples were reconstituted in 100 μl of 100% ethanol. The samples were detected and identified using by HPLC-MS/MS analysis.

### Scratch Wound Closure Assay

To assess the effect of AC on CAMs migration, we used the scratch wound assay as reported (Liang et al., 2007). 1.5 × 10^6^ of CAMs were cultured with DMEM containing 10% FBS for 1–2 days in a 3.5 cm cell culture dish. 100% confluent CAMs were scratched with a 200 ul sterile pipette tip. Then the dish was rinsed several times with PBS to remove the cells. After that, CAMs were cultured in DMEM containing 2% FBS and treated with different concentration of HMGB1 (0–1 ug/mL), Genistein (40 nM) or NOE (25 uM) for 24 h. Using an inverted microscope, cells migrated into the scratch were taken at 48 h. The results are presented as the percentage of wound healing, which was calculated as follows: Cell area/Wound area (initial) × 100.

### Cell Proliferation Assay

To verify the effect of AC on CAMs proliferation, WST-1 Kit was used to measure CAM proliferation. The CAMs were cultured at 0.5 × 10^4^ cells in 24-well plates containing in 10% FBS supplemented DMEM overnight. The next day, CAMs were changed with fresh 2% FBS supplemented DMEM. On day 5, the proliferation of CAMs was examed using WST- Kit. Cell culture medium was discarded and incubated with 200 ul of medium with WST-1 for 4–6 h in the 37°C incubator. The medium was then transferred to a 96-well plate to measure the absorbance (OD Value) at 450 nm. The data were expressed as ratios of the control value.

### Statistics

Data were presented as means ± SEM. Values were analyzed for significant differences of multiple groups using ANOVA for repeated measures followed by Duncan’s multiple range test. Significant differences between two groups of experiments were examined using the Student’s *T*-Test. SigmaPlot 13.5 software analysis (Systat Software, San Jose, CA, United States) was used to analyze statistical significance. Statistical significance was defined when *P* < 0.05.

## Results

### AC Expression and Its Upregulation or Downregulation in the Coronary Arterial Myocytes (CAMs)

**FIGURE 1:**
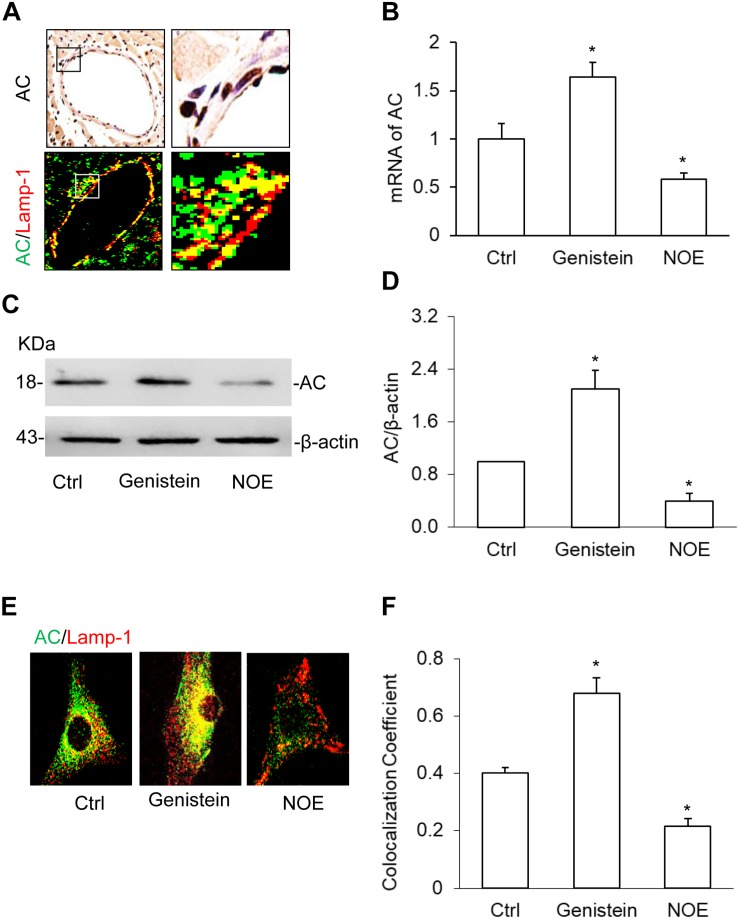
Acid ceramidase (AC) expression and it’s up- or downregulation in the coronary arterial myocytes (CAMs). **(A)** The representative images showing the expression of AC and the colocalization of AC with lysosome marker, lamp-1 using immunochemistry staining (IHC) and immunofluorescence staining in the coronary artery wall. **(B)** Summarized real-time PCR results showing the significant upregulation or downregulation of AC by genistein or N-oleoylethanolamine (NOE) in CAMs. **(C)** Representative Western blot gel documents showing the effects of genistein and NOE on the expression of AC in CAMs. **(D)** The summarized Western blot results showing the effects of genistein and NOE on the expression of AC in CAMs. **(E)** The representative immunofluorescence images showing the effects of genistein and NOE on the colocalization of AC with Lamp-1 in lysosomes. **(F)** The summarized data showing the effects of genistein and NOE on the colocalization of AC with lamp-1. Data are expressed as means ± SEM, *n* = 5, * *p* < 0.05 vs. Ctrl group.

There are few reports on the AC expression in SMCs; therefore, we first characterized AC expression and its upregulation or downregulation in CAMs. On the coronary arterial wall, we found AC protein expressed in the CAMs by immunochemistry staining and AC colocalized with lysosome marker, lamp-1 using immunofluorescence staining (Figure 1A). Using real-time PCR and Western blot, we further confirmed *Asah1* gene or AC protein expression in CAMs and its expression was increased by the inducer, genistein and decreased by the inhibitor, N-oleoylethanolamine (NOE) (Figures 1B–D). These results suggest that AC expression plays an important role in CAMs. We also discovered that genistein increased and NOE decreased the expression of AC in the lysosome by the colocalization of AC with lamp-1 in the primary culture CAMs (Figures 1E,F). These data indicate that AC may regulate the function of the lysosome.

### Effects of HMGB1 on AC Expression in CAMs

**FIGURE 2:**
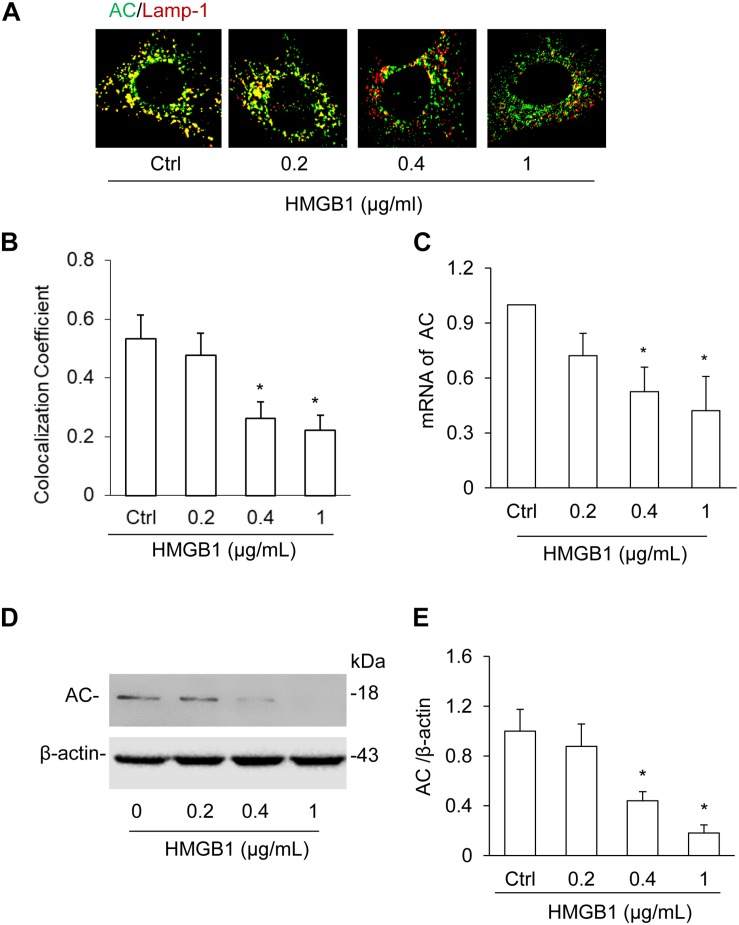
Effects of HMGB1 on AC expression in CAMs. **(A)** The representative images showing the effect of HMGB1 on the colocalization of AC with lamp-1 using immunofluorescence staining in CAMs. **(B)** Summarized graph showing the effect of HMGB1 on the colocalization of AC with lamp-1. **(C)** Summarized real-time PCR results showing the effect of HMGB1 on mRNA expression of *Aash1* gene in CAMs. **(D)** Representative Western blot analysis showing the effect of HMGB1 on the expression of AC in CAMs. **(E)** Summarized Western blot gel document showing the effect of HMGB1 on the expression of AC in CAMs. Data are expressed as means ± SEM, *n* = 5, *p < 0.05 vs. Ctrl group.

High-mobility group box 1 destabilizes the lysosomal membrane, which may affect AC maturation and expression in the lysosome (Andersson et al., 2018b). Using immunofluorescence microscopy, we confirmed whether HMGB1 treatment affects the interaction of AC with lysosomes in CAMs. Our summarized data showed that HMGB1 dose-dependently decreased the colocalization of AC with lamp-1 (Figures 2A,B), which demonstrate that HMGB1 affects AC function in the lysosome. Then real-time PCR and Western blot results also showed the dose-dependent decrease of AC expression as shown in Figures 2C–E. These results suggest that HMGB1 may be a key factor in regulating the AC function.

### HMGB1-Induced Downregulation of AC Expression in CAMs Treated With AC Gene Inducer or Inhibitor

**FIGURE 3:**
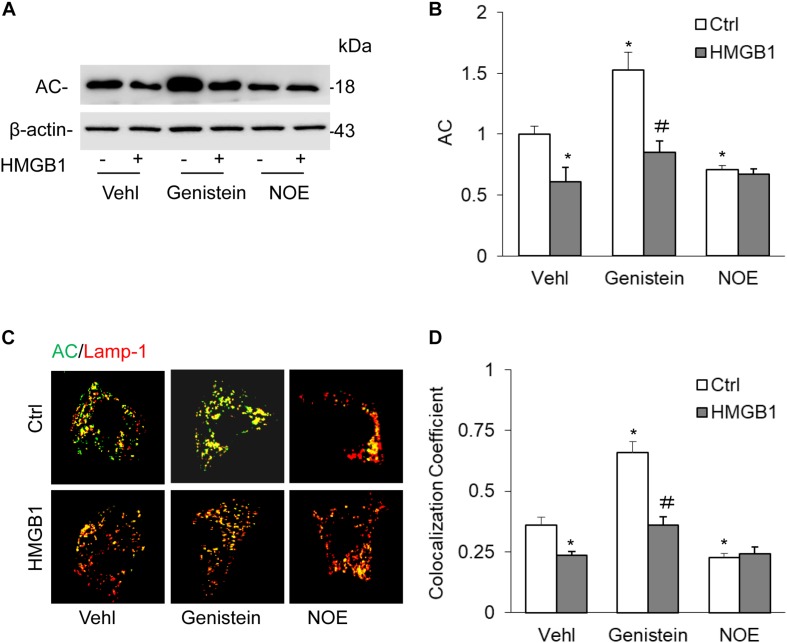
High-mobility group box 1-induced downregulation of AC expression in CAMs treated with AC gene inducer or inhibitor. **(A)** Representative Western blot gel documents showing the effect of HMGB1 on AC expression in CAMs with the treatment of genistein or NOE. **(B)** Summarized data showing the effect of HMGB1 on AC expression in CAMs s with the treatment of genistein or NOE. **(C)** The representative immunofluorescence images showing the effect of HMGB1 on the colocalization of AC with lamp-1 in CAMs treated with genistein or NOE. **(D)** Summarized data showing the effect of HMGB1 on the colocalization of AC with lamp-1 in CAMs treated with genistein or NOE. Data are expressed as means ± SEM, *n* = 5, * *p* < 0.05 vs. Vehl-Ctrl group, # *p* < 0.05 vs. Ctrl group.

To further confirm the effects of HMGB1 on AC expression, we studied the effects of HMGB1 on AC expression when treated with genistein and NOE. By Western blot analysis, we found that HMGB1 significantly attenuated AC expression induced by genistein. However, we did not discover any effects of HMGB1 on the decrease induced by NOE (Figures 3A,B). By detection of the colocalization of AC with lamp-1 using an immunofluorescence microscope, we also found genistein remarkably increased the colocalization of AC with lamp-1, but the increase was blocked by HMGB1 treatment in CAMs. Although NOE significantly decreased AC expression, we still did not see any further decrease when CAMs were treated with HMGB1 (Figures 3C,D).

### Effects of HMGB1 on Cellular Ceramide Levels in CAMs

**FIGURE 4:**
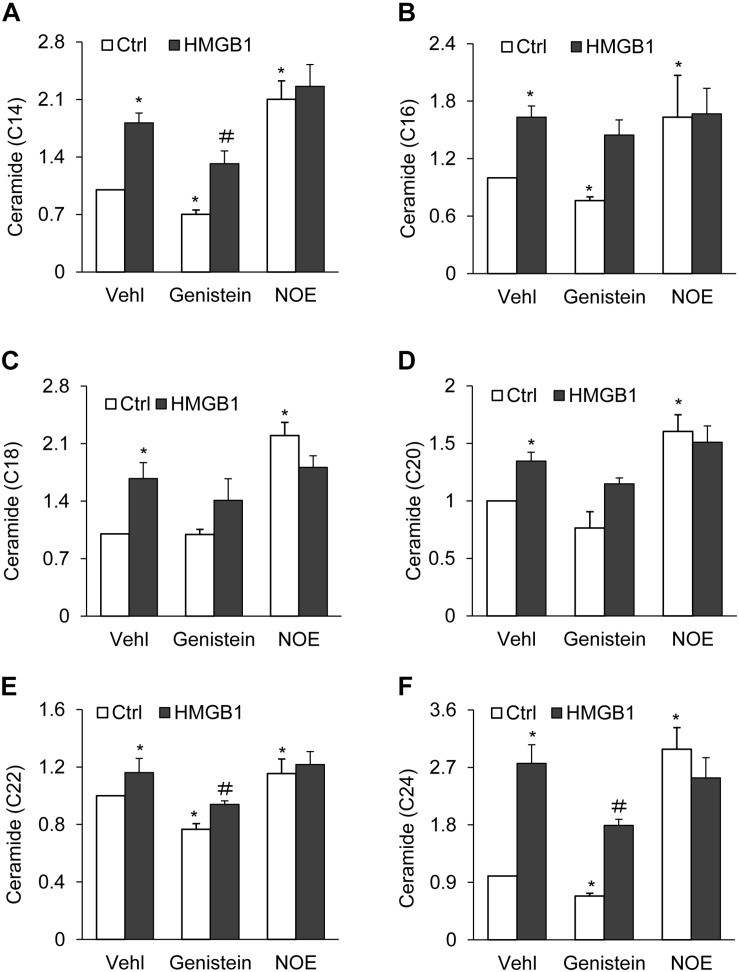
Effects of HMGB1 on cellular ceramide levels in CAMs. **(A–F)** Summarized LC-MS/MS analysis showing the effects of genistein and NOE on HMGB1 induced on CAMs ceramide accumulation. Data are expressed as means ± SEM, *n* = 5, * *p* < 0.05 vs. Vehl-Ctrl group, # *p* < 0.05 vs. Ctrl group.

It is known that ceramide can be hydrolyzed by AC to form both sphingosine and a free fatty acid; in addition, a deficiency of AC leads to an accumulation of ceramide (Yu et al., 2018b, 2019). Based on this knowledge, we quantified ceramide levels in CAMs by liquid chromatography-tandem mass spectrometry (LC-MS/MS). It was found that HMGB1 and NOE treatment significantly elevated C14, C16, C18, C20, C22, and C24, but genistein remarkably attenuated the increase of C14, C16, C22, and C24, except for C18 and C22, with or without HMGB1 treatment. We also observed that HMGB1 attenuated the increase C14, C22 as well as C24 induced by genistein, but HMGB1 did not further increase ceramide induced by NOE (Figures 4A–F).

### Effects of HMGB1 on Migration and Proliferation of CAMs

**FIGURE 5:**
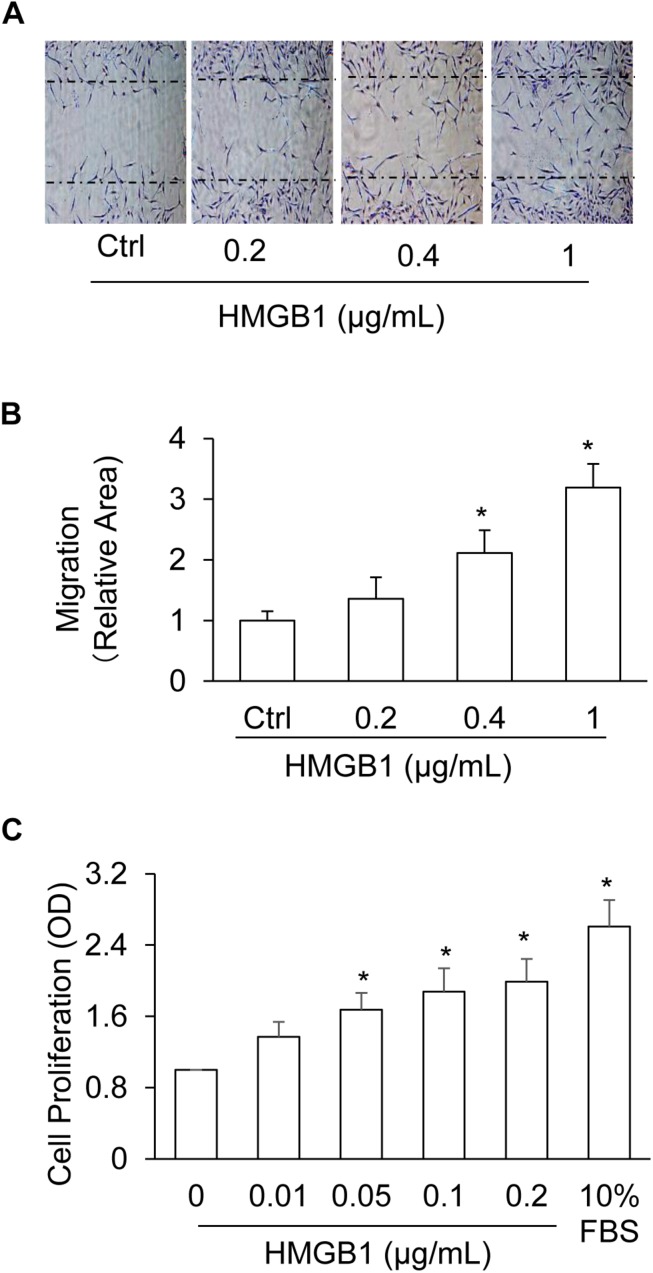
Dose-effect of HMGB1 on migration and proliferation of CAMs. **(A)** Representative wound healing assay images presenting the dose effects of HMGB1 on CAMs migration. **(B)** Summarized data showing the dose effects of HMGB1 on CAMs migration. **(C)** Summarized data showing the dose effects of HMGB1 on CAMs proliferation. Data are expressed as means ± SEM, *n* = 5, * *p* < 0.05 vs. Ctrl group.

Using wound healing assay and WST-1 Kit, we studied the effects of HMGB1 on the migration and proliferation of CAMs. As shown in Figures 5A,B, 0.4 ug/ml and 1 ug/ml of HMGB1 significantly increased CAMs migration. Interestingly, we found that a low concentration of HMGB1 (0.05–0.2 ug/ml) was promptly able to promote CAMs proliferation exceptionally (Figure 5C).

### Effects of AC Gene Induction and Inhibition on HMGB1-Induced Migration and Proliferation of CAMs

**FIGURE 6:**
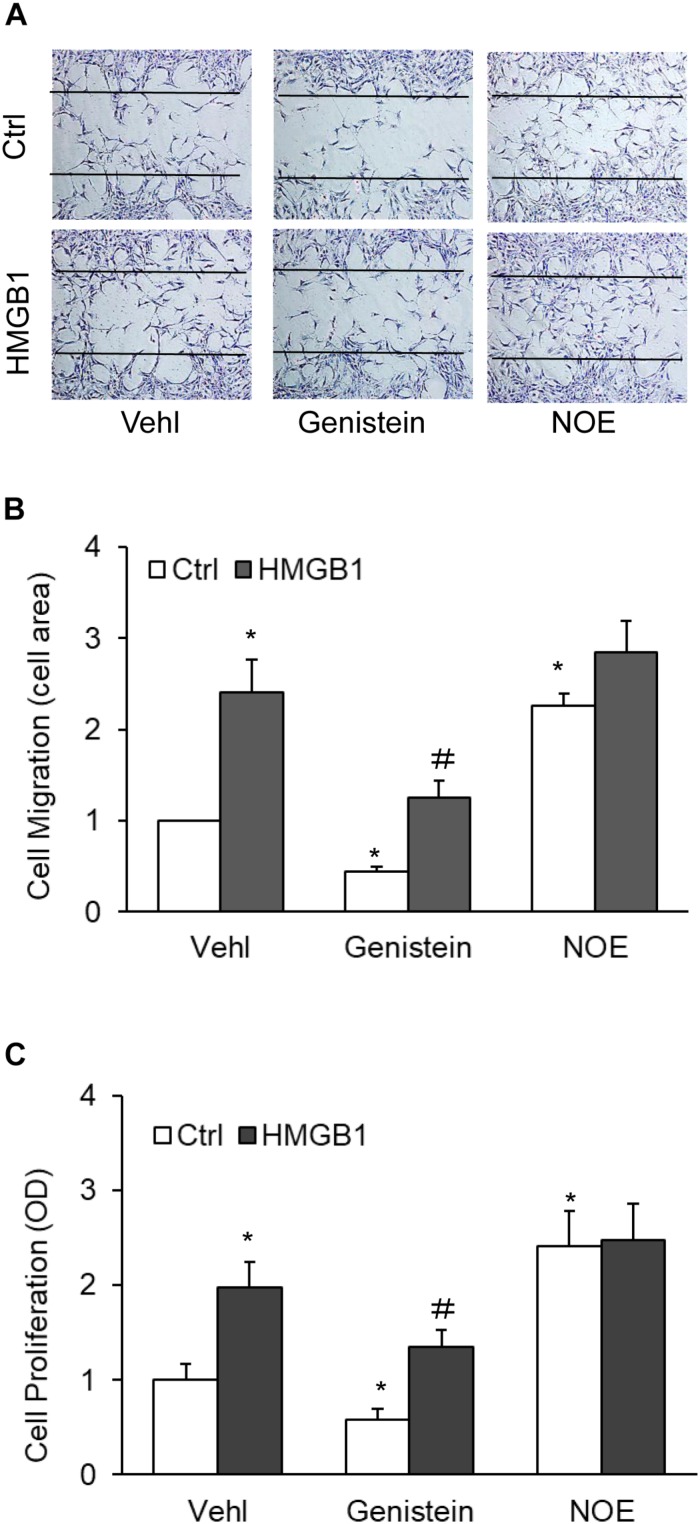
Effects of AC gene induction and inhibition on HMGB1-induced migration and proliferation of CAMs. **(A)** Representative wound healing assay images presenting the effect of AC induction and inhibition on HMGB1-induced migration. **(B)** Summarized data showing the effect of AC induction and inhibition on HMGB1-induced migration. **(C)** Summarized data showing the effect of AC induction and inhibition on HMGB1-induced CAMs proliferation. Data are expressed as means ± SEM, *n* = 5, * *p* < 0.05 vs. Vehl-Ctrl group, # *p* < 0.05 vs. Ctrl group.

To further study the effects of AC on the migration and proliferation in the CAMs, we next tested the effects of AC gene induction and inhibition on HMGB1-induced migration and proliferation of CAMs. As shown in Figures 6A,B, genistein, the AC gene inducer, significantly reduced CAMs migration and remarkably ameliorated CAMs migration induced by HMGB1. However, the inhibitor of the AC gene, NOE, increased CAMs migration, although there is no enhancement by HMGB1. We also tested the effects of genistein and NOE on HMGB1-induced proliferation and discovered similar results (Figure 6C). These results indicate that AC contributes to HMGB1-induced CAMs migration and proliferation.

### Effects of HMGB1 on Cellular Ceramide Levels in the CAMs From Mice With Asah1 Gene Deletion and Their Littermates

**FIGURE 7:**
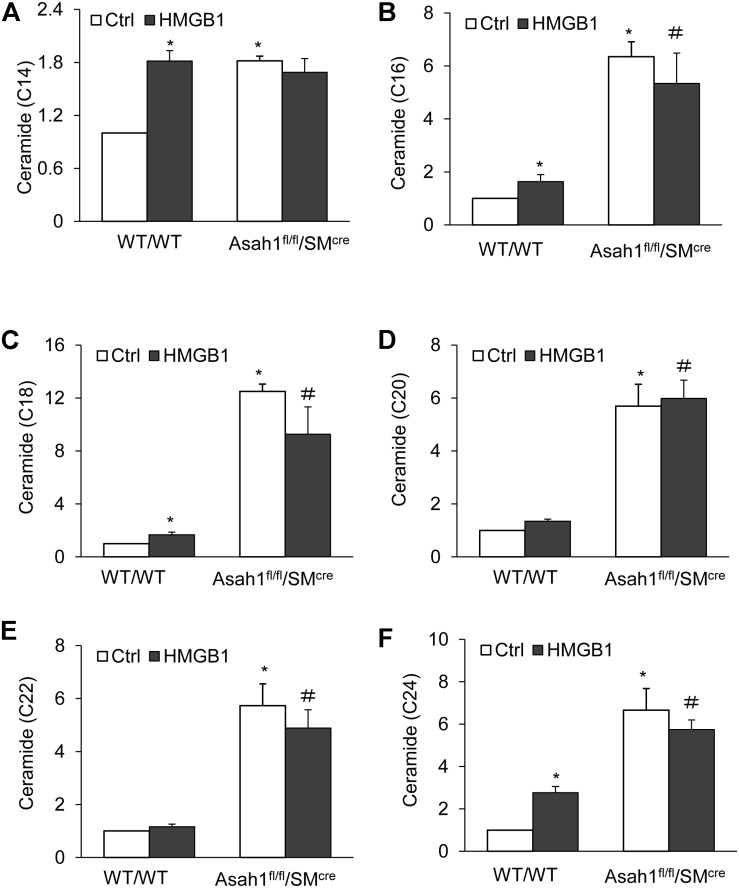
Effects of HMGB1 on cellular ceramide levels in CAMs from mice with *Asah1* gene deletion and their littermates. **(A–F)** Summarized LC-MS/MS analysis showing the effects of *Asah1* deletion on CAMs ceramide accumulation. Data are expressed as means ± SEM, *n* = 5, * *p* < 0.05 vs. WT/WT-Ctrl group, # *p* < 0.05 vs. WT/WT-HMGB1 group.

For additional insight into the effects of HMGB1 on the regulation mechanism of AC, we isolated in the CAMs from *Asah1* specific knock-out mice and measured the effects of HMGB1 on cellular ceramide levels in the CAMs from mice with *Asah1* gene deletion and their littermates. It was found that *Asah1* gene deletion significantly elevated C14, C16, C18, C20, C22, and C24, but HMGB1 treatment only, in comparison, produced a lesser increase of C16, C18, C20, C22, and C24 except for C14 (Figures 7A–F).

### HMGB1-Induced Migration and Proliferation in CAMs From Mice With Asah1 Gene Deletion and Their Littermates

**FIGURE 8:**
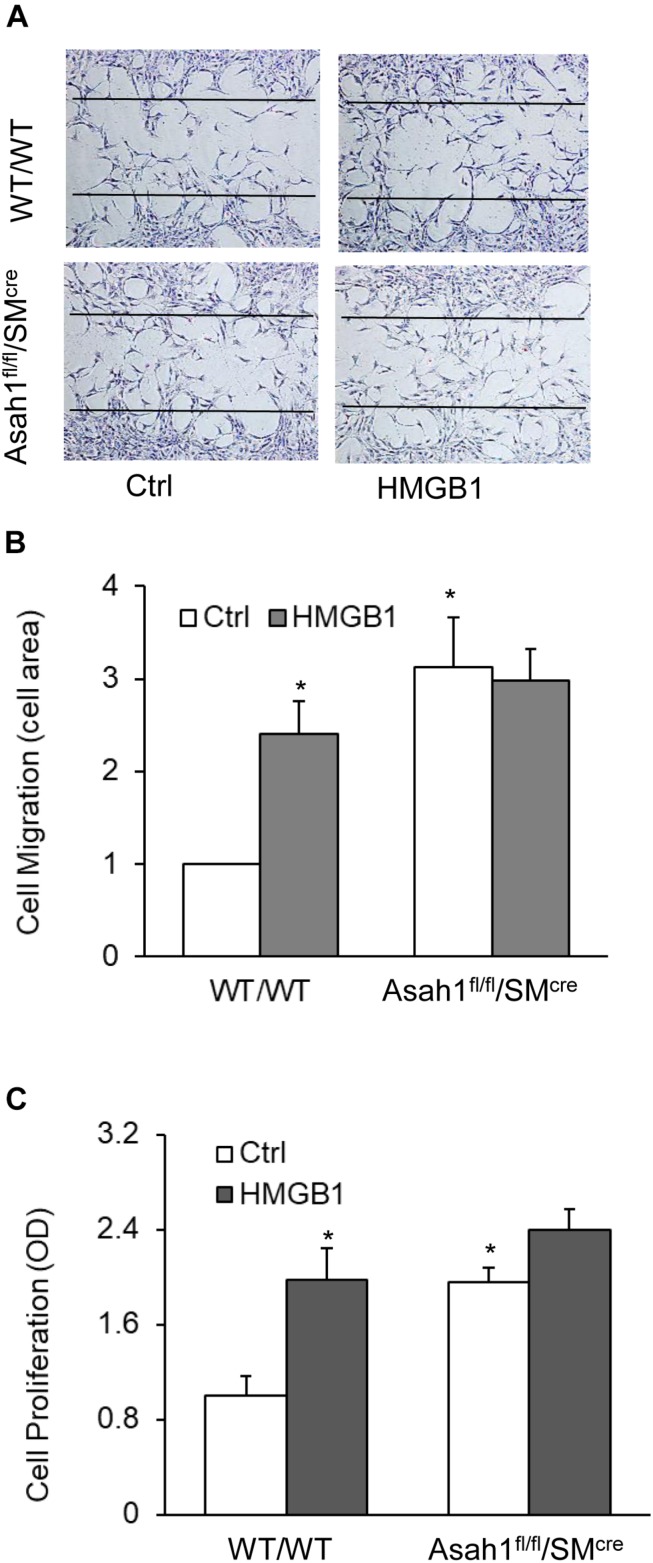
HMGB1-induced migration and proliferation in CAMs from mice with *Asah1* gene deletion and their littermates. **(A)** Representative wound healing assay images presenting the effect of AC deletion on CAMs migration. **(B)** Summarized data showing the effect of AC deletion on CAMs migration. **(C)** Summarized data showing the effect of AC deletion on SMCs CAMs proliferation. Data are expressed as means ± SEM, *n* = 5, * *p* < 0.05 vs. Ctrl group.

Finally, we demonstrated that HMGB1 treatment or *Asah1* gene deletion have similar effects on CAMs migration and proliferation. As shown in Figures 8A–C, *Asah1* gene deletion significantly increased CAMs migration and proliferation compared to control SMCs from WT mice. These data suggest the migration and proliferation induced by HMGB1 are mediated by *Asah1* gene.

## Discussion

The present study demonstrated a critical role of AC in HMGB1-induced CAMs migration and proliferation. Our study demonstrated that AC was expressed in CAMs and primarily localized in the lysosomes. HMGB1 suppressed the AC expression in CAMs, which was accompanied by increased ceramides, and enhanced migration and proliferation.

Acid ceramidase was first studied and partially purified by Gatt in 1963 (He et al., 2003). It was first purified to apparent homogeneity from urine as a ∼50-kDa polypeptide consisting of two subunits, α (∼13-kDa) and β (∼40-kDa) (Bernardo et al., 1995). AC is synthesized as a ∼55-kDa precursor that can be proteolytically processed into two subunits within late endosomes or lysosomes. AC expression was primarily found in cellular compartments, presumably in acidic organelles. Small amounts of AC precursors and a partially processed 47-kDa form of AC were also found extracellularly. Lysosomes are acidic organelles that contain approximately 50 different degradative enzymes active at acidic pH (4–5) (Mindell, 2012). Shtraizent reported that AC activation requires an autocatalytic cleavage at acidic pH, however, AC processing could also occur at neutral pH (Shtraizent et al., 2008). It has been suggested that AC is processed and activated in either lysosome or in other intracellular organelles. In the present study, we found that AC is expressed in the coronary artery wall of mice and in primary cultured CAMs. In CAMs, our confocal microscopic studies revealed that AC was distributed diffusely in a punctuated pattern throughout the cytosol and significantly colocalized with lysosome marker Lamp-1. These data suggest that AC may primarily reside in lysosomes but also localize in other non-lysosome organelles.

We further examined whether the pharmacological activation or inhibition of AC was well correlated with changes in the interaction of AC with Lamp-1. Genistein induces *Asah1* transcription through a GPR30-dependent, pertussis toxin-sensitive pathway that requires the activation of c-Src and extracellular signal-regulated kinase 1/2 (ERK1/2). Activation of this pathway promotes histone acetylation in addition to recruitment of phospho-estrogen receptor α and specificity protein-1 to the *Asah1* promoter, ultimately resulting in increased ceramidase activity (Lucki and Sewer, 2011). N-Oleoyl-ethanolamine (NOE), a well-established ceramidase inhibitor (Sugita et al., 1975), blocks ceramide deacylation to sphingosine in intact cells (Coroneos et al., 1995; Meacci et al., 1996; Wiesner and Dawson, 1996a, b; Oral et al., 1997). The present study demonstrated that genistein increased the AC expression and its interaction with Lamp-1 in CAMs (Figure 3), which was accompanied by decreased ceramide levels (Figure 4). Conversely, NOE decreased the AC expression and its interaction with Lamp1 (Figure 3), whereas ceramide levels were increased (Figure 4). Thus, these findings suggest that lysosomal AC plays a crucial role in modulating ceramide levels in CAMs.

Coronary arterial myocytes are the primary cell type in blood vessel walls and play essential roles in pathogenesis of vascular diseases. By transforming from the contractile to the synthetic phenotype, the CAMs exhibit distinct proliferative and migratory abilities and produce pro-inflammatory cytokines (Wang K. et al., 2017; Zhang et al., 2018). HMGB1 was demonstrated to stimulate cell proliferation and migration in a variety of mammalian cells including fibroblasts (Chitanuwat et al., 2013), airway SMCs (Hou et al., 2019), osteosarcoma cells (Guo et al., 2014), and cancer cells (He et al., 2018). Consistently, the present study for the first time revealed that HMGB1 promotes cell proliferation and migration in murine CAMs (Figure 5). We further demonstrated that HMGB1 downregulated AC expression and increased ceramides in CAMs, whereas HMGB1-induced AC inhibition and ceramide accumulation were attenuated by AC activator genistein. HMGB1 were demonstrated to bind cell surface receptors including RAGE, TLR2, TLR4, and TLR9 (Tsung et al., 2014; Xu et al., 2014; Kim et al., 2018). The molecular mechanisms by which these receptors mediate AC inhibition by HMGB1 deserve further elucidation. Moreover, AC inhibitor NOE mimicked the effects of HMGB1 on CAM proliferation and migration, whereas these effects by HMGB1 were attenuated by AC activator genistein. To our knowledge, our findings provide the first evidence that HMGB1-induced CAM phenotypic switching is associated with AC inhibition and ceramide accumulation.

In recent years, many studies have revealed the correlation of circulating ceramide levels with cardiovascular events such as myocardial infarction and stroke (Summers, 2018; Tippetts et al., 2018). However, the molecular mechanisms by which certain ceramides drive cardiovascular dysfunction remains largely unknown. It has been shown that ceramides with long chains (e.g. C16:0, C18:0, C20:0, C22:0, C24:1) are associated with deleterious outcomes, independent of plasma lipid markers and other cardiovascular risk factors (Tarasov et al., 2014; Havulinna et al., 2016; Wang D.D. et al., 2017; Anroedh et al., 2018; Meeusen et al., 2018). In mice, HMGB1 injections were noted to increase ceramides in muscle cells, especially C16 and C24 ceramides, enhance reactive oxygen species (ROS) production, and disrupt mitochondrial and insulin function, effects were alleviated with ceramide inhibition (Taylor et al., 2017). The present study also demonstrated that HMGB1 and NOE similarly increased long-chain ceramides including C14, C16, C18, C20, C22, and C24 ceramides. Collectively, these findings suggest that the injurious effects of HMGB1 on the vasculature may be associated with AC inhibition and consequent accumulation of long-chain ceramides in CAMs.

Finally, we examined the effects of AC on CAM proliferation and migration using primary cultures of CAMs from the mice with *Asah1* gene deletion and their littermates. Our previous studies have shown that *Asah1* gene deletion induced a phenotypic change in phosphate (Pi)-treated CAMs, as depicted by decreased SM22-α (SMC marker) expression (Bhat et al., 2018). In the present study, we demonstrated that AC gene deficiency increased the migration and proliferation in CAMs, an effect resembled by the HMGB1 or NOE. Thus, these data further support the view that downregulation of AC contributes to HMGB1-induced phenotypic switching in CAMs leading to increased migration and proliferation. The present study did not attempt to identify the signaling pathway(s) or mediators downstream of the HMGB1-AC-ceramides axis that acts on the proliferation and migration of SMCs. Recent studies demonstrated that both HMGB1 and ceramides can either stimulate or regulate intracellular signaling pathways/mediators such as ROS, NO, calcium signaling, MAPK signaling, AMPK/mTOR signaling, and lysosome destabilization (Chitanuwat et al., 2013; Guo et al., 2014; Xu et al., 2014; He et al., 2018; Hou et al., 2019). These signaling pathways/mediators have been involved in the regulation of proliferation and migration in mammalian cells including SMCs (Xu et al., 2014). Therefore, the crosstalk between ceramide and these intracellular signaling pathways/mediators may contribute to the HMGB1 effect on CAM proliferation and migration. In addition, a recent study reported that HMGB1 increases IL-1β release in vascular SMCs through NLRP3 inflammasomes (Kim et al., 2018). Inflammatory cytokines such as IL-1β was known to promote proliferation and migration of SMCs (Libby et al., 1988; Yamamoto et al., 1998). Thus, it is plausible that inflammation *per se* also contributes to HMGB1-induced CAM migration and proliferation. Future investigation is needed to explore the precise mechanism by which the HMGB1-AC-ceramide axis triggers the CAM phenotypic switching.

In summary, the present study demonstrated that AC alters cellular ceramide levels of CAMs and that AC may be a signaling enzyme mediating the action of HMGB1 in the regulation of CAM phenotypes in response to atherogenic or inflammatory stimuli. Indeed, recent studies have demonstrated that HMGB1 is increased in the arterial wall of mice fed with high-fat diet or mice with hyperglycemia/hypercholesterolemia (Elizondo-Vega et al., 2015; Zhang et al., 2015; Chen et al., 2016; Wang et al., 2016). Thus, it is speculated that the effect of HMGB1 on CAM phenotype switching through AC inhibition contributes to the pathogenesis of vasculopathy associated with metabolic disorders such as obesity and diabetes. Our findings also provide novel insights into understanding the therapeutical intervention for preventing vascular remodeling through targeting HMGB1 signaling axis.

## Data Availability Statement

The raw data supporting the conclusions of this manuscript will be made available by the authors, without undue reservation, to any qualified researcher.

## Author Contributions

XY participated in the research design, conducted the experiments, performed the data analysis, and wrote the manuscript. OB conducted the experiments and wrote the manuscript. HL generated the knock-out mouse and conducted the writing of manuscript. YZ and P-LL participated in the research design, performed the data analysis, and contributed to the writing of the manuscript. All authors contributed to the manuscript revision, read and approved the submitted version of the manuscript.

## Conflict of Interest

The authors declare that the research was conducted in the absence of any commercial or financial relationships that could be construed as a potential conflict of interest.
